# Biosynthesis, characterization, and biofunctional assessment of niobium oxide nanostructures obtained using lactic acid bacteria-derived supernatants

**DOI:** 10.3762/bjnano.17.77

**Published:** 2026-08-17

**Authors:** Pablo Araujo Oliveira, Gabriella T L Teixeira, Pollyane S Oliveira, Thaís S Farnesi-de-Assunção, Isabela S Rotta, Karina F D Vicentine, Dayane S Alvares, Jéferson A Moreto, Aline D Paiva, Natália Bueno Leite Slade

**Affiliations:** 1 Institute of Exact Sciences, Naturals and Education, Federal University of Triângulo Mineiro, Uberaba, 38064-200, Minas Gerais, Brazil; 2 Institute of Biological and Natural Sciences, Federal University of Triângulo Mineiro, Uberaba, 38025-350, Minas Gerais, Brazil; 3 Institute of Biosciences, Letters and Exact Sciences, São Paulo State University, São José do Rio Preto, 15054-000, São Paulo, Brazil; 4 Department of Materials Engineering, São Carlos School of Engineering, University of São Paulo, São Carlos, 13566-590, São Paulo, Brazil

**Keywords:** antimicrobial activity, green synthesis, *Lacticaseibacillus paracasei*, *Lacticaseibacillus rhamnosus*, niobium oxide nanoparticles

## Abstract

Nanotechnology offers innovative opportunities for developing functional materials with applications across biomedical and food-related fields. Among metallic and metal oxide nanostructures, niobium-based compounds have recently attracted attention due to their remarkable mechanical strength, corrosion resistance, thermal conductivity, and biocompatibility. In this study, niobium oxide nanostructures were synthesized through a green, cell-free approach using supernatants derived from lactic acid bacteria (LAB) *Lacticaseibacillus rhamnosus* and *Lacticaseibacillus paracasei* UFTM 2.9. The use of LAB supernatants provided an eco-friendly and sustainable route for nanoparticle production, avoiding toxic reagents while exploiting biologically active metabolites for bioreduction and stabilization. The synthesized nanostructures were characterized by scanning electron microscopy coupled with energy-dispersive X-ray spectroscopy (SEM/EDX), scanning-transmission electron microscopy (STEM), ultraviolet–visible spectroscopy (UV–vis), Fourier transform infrared spectroscopy (FTIR), and X-ray diffraction (XRD) and zeta potential analysis. In vitro assays using Vero-CCL81 cells demonstrated that the nanostructures exhibited low cytotoxicity, confirming their potential biocompatibility. Antibacterial and antibiofilm activities were evaluated against *Escherichia coli* ATCC 25922, revealing significant inhibitory effects. These findings highlight a novel and sustainable route for producing niobium oxide nanostructures via LAB-mediated synthesis, with promising implications for biomedical and food safety applications.

## Introduction

Nanotechnology is a field focused on the study, development, and application of materials at the nanoscale [1–2]. Among the various types of nanomaterials, metallic and metal oxide nanostructures have stood out due to their distinctive physicochemical and biological properties, which make them promising for applications in the biomedical field. In this context, nanoparticles composed of silver, gold, iron, and selenium have received considerable attention [2–3].

Niobium stands out for its remarkable combination of properties, such as high mechanical strength, corrosion stability, efficient thermal conductivity, and excellent biocompatibility, which make it attractive for a wide range of technological applications [4–6]. Additionally, Brazil is the leading global holder of niobium resources, accounting for approximately 98% of the world’s known reserves [4]. Furthermore, niobium-based nanostructures represent a viable strategy to expand the applications of the metal beyond its commonly used forms [6–8].

To fully exploit the potential of niobium’s properties in nanoscale applications, it is essential to adopt sustainable and environmentally friendly synthesis methods. In this context, green synthesis has emerged as a promising approach for the production of nanostructures, providing a safer and more ecological alternative to conventional physical and chemical routes, primarily by avoiding the use of toxic and high-cost substances [9–10]. Among the resources considered for use in the green synthesis of nanoparticles are extracts derived from lactic acid bacteria (LAB), a group of microorganisms widely used in different industrial processes, precisely because of their ability to synthesize compounds with different biological activities [11–13]. Supernatants obtained from LAB cultures exhibit a pool of metabolites whose functional groups can drive the biosorption and bioreduction processes, essential for the synthesis of metallic nanostructures [12–14]. The use of these metabolites in the synthesis of metallic and metal oxide nanostructures represents a promising strategy for the development of new functional materials [13,15].

The efficiency and potential of these processes have been demonstrated by recent studies exploring the use of *Lacticaseibacillus rhamnosus* (*Lcr*) and *Lacticaseibacillus paracasei* as biological agents in the synthesis of metallic nanostructures. Lavecchia et al. demonstrated that the synthesis of silver nanoparticles using *Lcr* resulted in a material with strong antimicrobial activity and high photocatalytic efficiency [16]. Similarly, Awadelkareem et al. reported that silver nanoparticles synthesized using *Lcr* exhibited promising antimicrobial activity and the ability to inhibit biofilm formation by pathogenic bacteria, suggesting potential applications in biomedical and therapeutic fields [17]. Zeinivand et al. reported that the synthesis of silver nanoparticles mediated by *Lacticaseibacillus paracasei* resulted in compounds with effective activity against pathogenic microorganisms, including Gram-positive and Gram-negative bacteria as well as the yeast *Candida albicans*, also highlighting their inhibitory potential in biofilm formation [18].

Previous studies conducted by our research group demonstrated that *Lacticaseibacillus paracasei* UFTM 2.9 (*Lcp*), a strain isolated from raw milk, exhibited key functional attributes, such as survival of in vitro exposure to acidic pH and bile salts, antimicrobial activity against potential pathogens, and an antimicrobial susceptibility profile compatible with probiotic candidacy [19]. In parallel, we have also investigated the biological potential of niobium-based nanostructures, which exhibit an antibacterial and antibiofilm activity against *Escherichia coli* [6]. Furthermore, the incorporation of organic compounds has proven effective in enhancing the biocompatibility of the material while preserving its bioactive properties [6].

In view of these aspects, the aim of this study was synthesis, characterization, and biological evaluation of niobium oxide nanostructures produced using cell-free supernatants (CFSs) of *Lc. rhamnosus* and *Lc. paracasei* UFTM 2.9. Morphological and structural characterization techniques were employed, including scanning electron microscopy coupled with energy-dispersive X-ray spectroscopy (SEM/EDX), scanning-transmission electron microscopy (STEM), ultraviolet–visible spectroscopy (UV–vis), Fourier transform infrared spectroscopy (FTIR), and X-ray diffraction (XRD). Furthermore, the surface charge properties were determined by zeta potential measurements. In vitro biological assays were performed using Vero-CCL81 cell cultures to evaluate the cytotoxic effects. The antibacterial and antibiofilm activities were evaluated using *Escherichia coli* ATCC 25922 as a target microorganism. The results presented herein demonstrate an innovative research approach for the synthesis of niobium oxide-based nanostructures using bacterial supernatants. This strategy not only enables the development of synergistic materials by combining the bioactive properties of LABs, but also highlights their potential for biomedical applications.

## Results and Discussion

**Figure 1:**
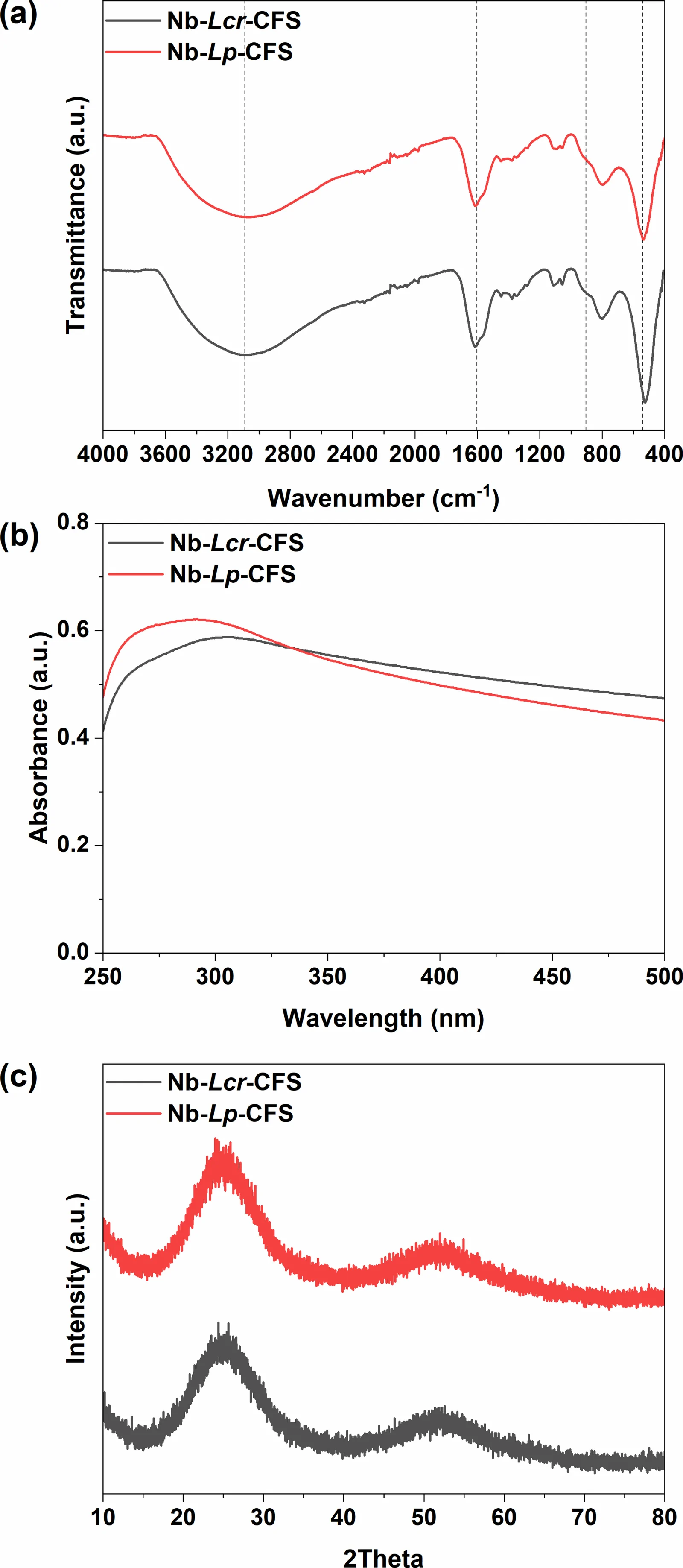
Structural and optical characterization of niobium-based materials synthesized in the presence of *Lcr*-CFS and *Lcp*-CFS: (a) FTIR spectra; (b) UV–vis absorption spectra; (c) XRD patterns.

The niobium-based nanostructures synthesized in the absence of LAB-CFS (Nb-nano) used in this study were previously characterized in detail by our group, and their full structural, optical, morphological, elemental, and zeta potential characterization has already been published in Oliveira and coworkers [6]. The functional groups present in the niobium-based nanostructures synthesized using LAB cell-free supernatants were analyzed by FTIR spectroscopy, as shown in Figure 1a. Both Nb-*Lcp*-CFS and Nb-*Lcr*-CFS samples exhibited characteristic absorption bands around 3075, 1614, 800, and 530 cm^−1^, which can be attributed to intrinsic vibrational modes of the inorganic niobium oxide framework [6]. The features near 3075 cm^−1^ [20] and 1614 cm^−1^ [21] correspond to the stretching and bending vibrations of O–H groups associated with molecularly adsorbed water, suggesting the presence of surface hydroxylation. Absorptions typically observed around 900 cm^−1^, assigned to Nb=O stretching [6,22–23], were slightly shifted to lower wavenumbers (≈800 cm^−1^). This shift implies stronger interactions between Nb=O bonds and protonic species (H^+^) [24], likely mediated by functional biomolecules remaining from the supernatants. Such interactions indicate that the biomolecular environment not only influences oxide hydration but may also contribute to surface modification of the nanostructures through weak coordination or hydrogen bonding. The additional band detected near 530 cm^−1^ is consistent with Nb–O stretching vibrations [21]. Altogether, the spectral profile supports the formation of hydrated niobium oxide as the predominant phase [6,22,25], whose structural and chemical features appear to be subtly modulated by the bioactive compounds present in the CFS.

In addition to identifying the inorganic matrix, FTIR spectroscopy also provides valuable insight into the organic components originating from the *Lacticaseibacillus*-derived cell-free supernatants. These biomolecules, primarily fatty acids, proteins, and polysaccharides, can act as reducing, stabilizing, and functionalizing agents during the synthesis of metal oxides [26]. Table 1 summarizes the main functional groups corresponding to the vibrational bands detected in the spectra. In both materials synthesized in the presence of LAB, absorption bands located around 1446 cm^−1^ are assigned to C–H stretching vibrations of methylene groups, typical of proteinaceous compounds [15,26–28]. The bands detected near 1284 and 1100 cm^−1^ are attributed to C–N stretching vibrations associated with aromatic and aliphatic amines, respectively [2,26,28], indicating the incorporation of amino acid residues and peptide linkages from bacterial metabolites.

**Table 1:** FTIR absorption bands of materials in the presence of LAB and their corresponding functional groups.

Spectrum band (cm^−1^)		Functional group	References

Nb-*Lcp*-CFS	Nb-*Lcr*-CFS		

3075	3079	O–H (stretching)	[20]
1446	1446	C–H (alkane)	[15,26–28]
1378	1378	O–H (phenol) C–H (aromatic and aliphatic amine)	[15] [29]
1284	1284	C–N (aromatic amine)	[2,26,28]
1091	1113	C–N (aliphatic amine)	[2,26,28]
1058	1058	S=O	[15]
1614	1614	O–H (bending)	[21]
797	803	Nb=O	[6,22–24]
536	524	Nb–O	[21–22]

An additional feature around 1058 cm^−1^ can be related to sulfoxide (S=O) stretching [15], suggesting the presence of oxidized sulfur-containing biomolecules, possibly derived from cellular enzymes or thiol-bearing peptides. Moreover, the absorption observed near 1378 cm^−1^ is associated with various organic functional groups, further confirming the contribution of biogenic macromolecules to the surface chemistry of the nanostructures [15,29].

Collectively, these findings indicate that metabolites derived from *Lacticaseibacillus rhamnosus* and *Lacticaseibacillus paracasei* UFTM 2.9 were successfully incorporated or adsorbed onto the niobium oxide surface. The coexistence of inorganic (Nb–O, Nb=O) and organic (C–H, C–N, S=O) vibrational bands suggest a hybrid interface formed through green synthesis, in which biomolecular residues contribute to surface functionalization and potentially enhance biocompatibility and antifouling properties of the resulting nanostructures.

The spectrophotometric properties of the synthesized nanostructures were evaluated by UV–vis spectroscopy. Figure 1b shows the spectral behavior of the materials at a final concentration of 0.37 mg·mL^−1^ in an aqueous medium containing 1% (v/v) Tween 80. The samples were obtained by aliquoting a 2 mg·mL^−1^ stock solution, previously sonicated and dispersed prior to use. This approach was employed to optimize the solubility and dispersion of the nanostructures, a strategy previously applied to other lowly soluble metal oxides such as copper and zinc oxides [30–31]. The Nb-*Lcp*-CFS material exhibited an absorbance peak near 292 nm, whereas Nb-*Lcr*-CFS displayed a maximum around 306 nm. This spectral pattern is consistent with literature reports for Nb_2_O_5_-based materials [32–33], as well as with previous studies showing that niobium nanostructures synthesized without the addition of organic compounds exhibit a maximum absorbance around 310 nm [6]. The presence of smaller particles can induce a blueshift of the maximum absorbance [32], an effect observed for Nb-*Lcp*-CFS. Additionally, morphological variations, including particle size and shape, may contribute to spectral changes [34]. Although metabolites present in *Lcr*-CFS and *Lcp*-CFS were detected by FTIR analyses (Figure 1a), their presence did not result in significant changes in the optical properties of the nanostructures. For comparison purposes, the UV–vis spectra of the control niobium nanostructures synthesized in the absence of LAB-CFS (Nb-nano) and the isolated LAB-CFS samples were also evaluated (Supporting Information File 1, Figure S1). The control Nb-nano exhibited the characteristic absorption profile previously reported for hydrated Nb_2_O_5_ nanostructures [6], while the isolated supernatants displayed distinct spectral features associated with their biomolecular composition. However, because LAB-CFS represented only 10% of the reaction volume during synthesis and the resulting nanostructures were extensively washed prior to characterization, a direct spectral correlation between the purified nanostructures and the original supernatants is not expected. Nevertheless, these control analyses provide additional qualitative support for the formation of LAB-CFS-mediated niobium nanostructures. Accordingly, the UV–vis spectra confirm the successful formation of the niobium-based nanostructures described herein, while preserving the intrinsic optical features of hydrated Nb_2_O_5_.

XRD measurements were performed to assess the crystallinity of the synthesized materials, and the corresponding diffraction patterns are shown in Figure 1c. The nanostructures synthesized in the presence of *Lcp*-CFS and *Lcr*-CFS exhibited very similar diffraction profiles, characterized by two broad peaks centered at approximately 25° and 52°. The absence of sharp reflections is consistent with the formation of low crystalline or amorphous hydrated Nb_2_O_5_ [6,35–38] and may also reflect the presence of ultrasmall domains [22]. These findings agree with previous results from our research group on niobium-based nanostructures [6]. Taken together, the XRD analyses indicate that all obtained materials correspond to amorphous niobium pentoxide, with no detectable structural differences arising from the specific LAB-derived supernatant used during synthesis.

**Figure 2:**
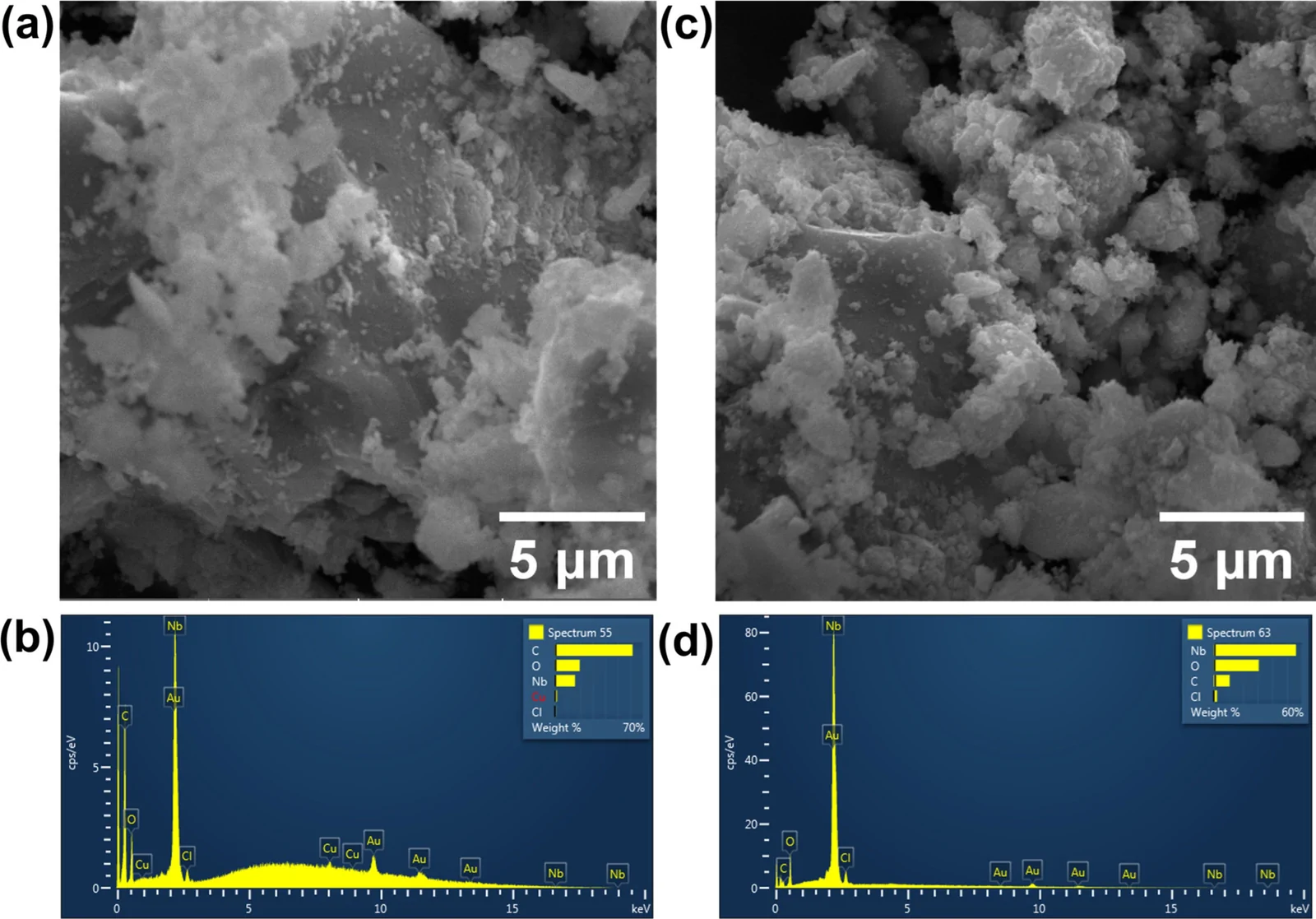
Representative SEM images of (a) Nb-*Lcr*-CFS and (c) Nb-*Lcp*-CFS at 10,000× magnification. EDX spectra of (b) Nb-*Lcr*-CFS and (d) Nb-*Lcp*-CFS. The gold (Au) signal arises exclusively from the Au coating deposited during sample preparation by sputtering.

The morphology of the synthesized nanostructures was investigated via SEM for Nb-*Lcr*-CFS (Figure 2a) and Nb-*Lcp*-CFS (Figure 2c). The micrographs revealed aggregates of irregular and poorly defined particles, with no apparent influence of the specific LAB supernatant used during biosynthesis. This morphological pattern aligns with previous observations of niobium-based nanostructures [6] and with reports on Nb_2_O_5_·*n*H_2_O-derived materials [38]. It is also worth noting that the inherently low conductivity of Nb_2_O_5_ [39] may contribute to the limited resolution typically obtained in SEM imaging of these samples.

EDX analyses were performed to determine the chemical composition of Nb-*Lcr*-CFS (Figure 2b) and Nb-*Lcp*-CFS (Figure 2d). Although the SEM images did not reveal substantial morphological variations, the elemental composition of the nanostructures varied according to the LAB supernatant used during biosynthesis. Samples synthesized with *Lcr*-CFS exhibited a markedly higher carbon content, whereas those obtained with *Lcp*-CFS displayed comparatively lower levels of this element, as shown in Table 2. This difference may reflect distinct metabolic profiles between *Lc. rhamnosus* and *Lc. paracasei*, which are known to release biomolecules such as organic acids, peptides, and residual metabolites [26]. Such species can adsorb onto the surface of forming nuclei or become partially incorporated during growth, contributing to carbon enrichment. In all cases, the detection of niobium and oxygen confirms that the predominant inorganic phase corresponds to niobium oxides.

**Table 2:** Elemental composition of the synthesized materials obtained by EDX analysis.

Element	Mass fraction (wt %)	

	Nb-*Lcr*-CFS	Nb-*Lcp*-CFS

Nb	16.01	56.16
C	61.84	10.83
O	19.79	30.77
Cl	0.67	2.24
Cu	1.69	—

The copper signal can be attributed to the tape used for sample fixation during SEM analysis, whereas the detection of chlorine likely results from residual precursor material that remained after washing steps. The carbon content observed in the samples aligns with the findings of Harandi et al., who reported significant incorporation during the biosynthesis of zinc oxide nanoparticles using *Lactobacillus plantarum* and *L. acidophilus*. According to the authors, such variations in elemental composition may arise from differences in bacterial metabolic activity and biomolecule release in the culture medium [11]. Thus, the EDX data support the FTIR observations, confirming the formation of niobium oxides and indicating the incorporation of LAB-derived metabolites into synthesized nanostructures.

**Figure 3:**
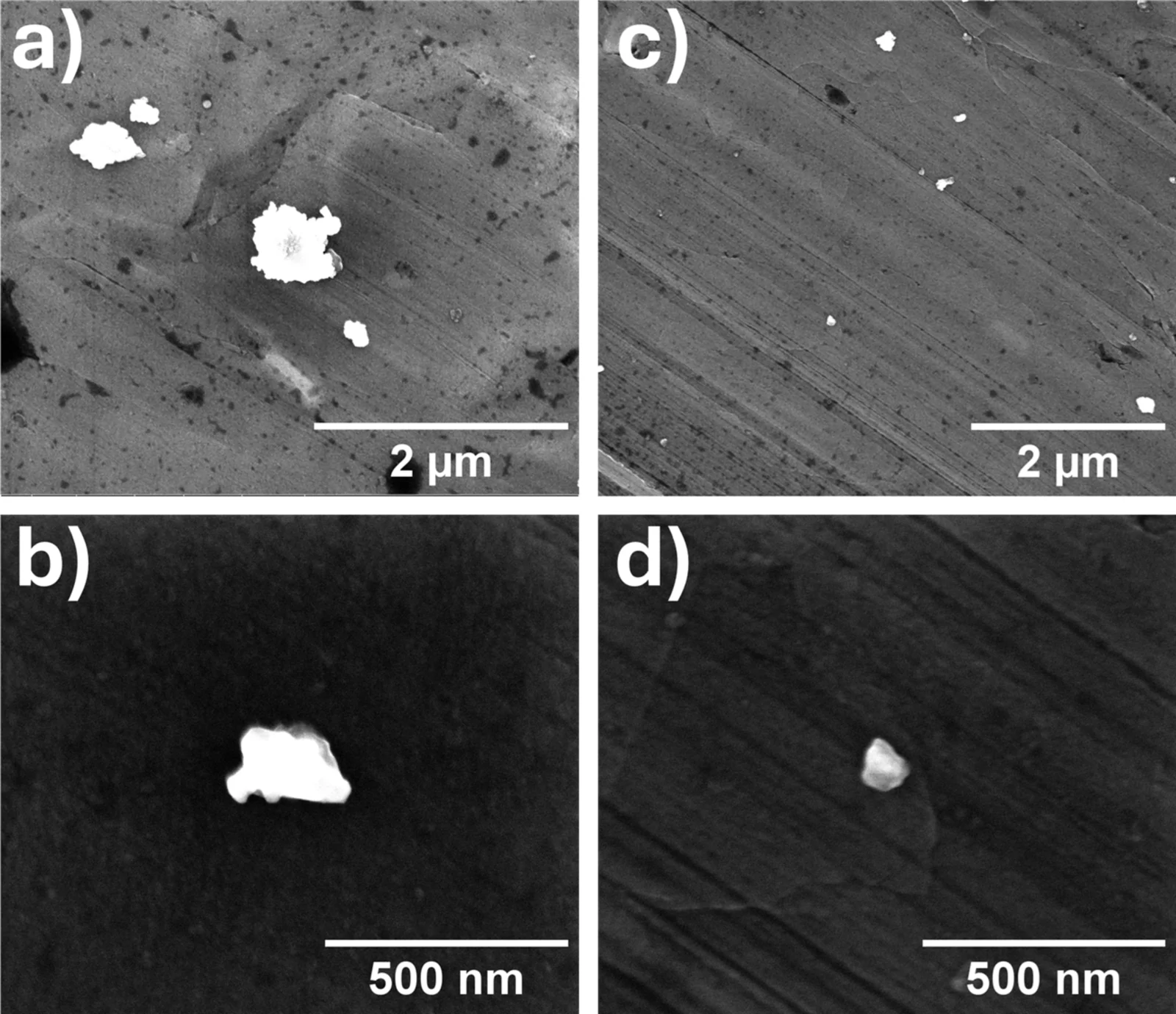
Morphological characteristics of the synthesized nanostructures obtained via STEM: (a,b) Nb-*Lcr*-CFS, and (c,d) Nb-*Lcp*-CFS.

To complement the morphological characterization obtained by SEM, the niobium-based nanostructures synthesized in the presence and absence of cell-free supernatants were further analyzed by STEM, as shown in Figure 3. In all samples, the images reveal the formation of clusters with irregular morphology and no well-defined geometric shape, reinforcing the observation that the biosynthetic conditions did not markedly alter the nanoscale morphology. Even at the highest magnification, as shown in Figure 3d for Nb-*Lcp*-CFS, the presence of aggregated clusters remains evident. These morphological characteristics are consistent with previous studies in the literature on the synthesis of niobium-based nanostructures [6,22,36–37]. Furthermore, the clusters exhibited average diameters of approximately 90 nm for Nb-*Lcp*-CFS and 255 nm for Nb-*Lcr*-CFS, indicating that the nature of the LAB supernatant influences the extent of particle aggregation. Aggregate sizes for Nb-*Lcr*-CFS and Nb-*Lcp*-CFS were estimated from STEM micrographs acquired at 250,000× magnification (Figure 3b and Figure 3d, respectively) using ImageJ software, as these images provided the highest contrast and aggregate discrimination. However, due to the pronounced aggregation of the nanostructures and the extensive overlap between adjacent particles, reliable segmentation of individual particles was not possible, precluding the acquisition of a statistically representative particle size distribution, including standard deviations and size histograms. Therefore, the reported values should be regarded as approximate aggregate size estimates intended for comparative assessment between samples. In this context, STEM was employed as a complementary morphological characterization technique rather than as a comprehensive statistical particle size analysis.

To complement the morphological characterization, dynamic light scattering (DLS) measurements were performed in aqueous medium. Nb-*Lcp*-CFS exhibited a hydrodynamic diameter of 443 ± 31 nm with a polydispersity index (PDI) of 0.11, whereas Nb-*Lcr*-CFS showed a hydrodynamic diameter of 585 ± 67 nm and a PDI of 0.28. Based on the SEM and STEM results, these values are likely associated with nanoparticle agglomerates rather than individual particles, reflecting the tendency of these nanostructures to aggregate in aqueous suspension. Similar behavior has been reported by Cao et al. [40], who observed that zinc oxide nanoparticles with different morphologies and particle sizes ranging from approximately 7 to 49 nm, as determined by transmission electron microscopy (TEM), exhibited hydrodynamic diameters between 526 and 881 nm in water, even after sonication to minimize aggregation effects. Therefore, the hydrodynamic sizes obtained for the niobium-based nanostructures green-synthesized with *Lcp*-CFS and *Lcr*-CFS do not directly correspond to the particle dimensions observed by electron microscopy, but rather to the size of the aggregates formed in the aqueous medium.

Zeta potential is widely recognized as an important parameter for evaluating the surface charge of nanomaterials and providing insight into their colloidal behavior [41]. In the present study, niobium-based nanostructures synthesized in the presence of LAB-derived metabolites exhibited zeta potential values of −15.0 ± 0.7 mV (Nb-*Lcr*-CFS) and −13.1 ± 0.3 mV (Nb-*Lcp*-CFS). For comparison, Oliveira et al. reported a value of −17.6 mV for nanostructures synthesized in the absence of LAB-derived metabolites [6], indicating a slight variation in surface charge upon the use of bacterial supernatants. Similar effects have been described by Solís-Sandí et al., who demonstrated that biomolecules present in bacterial extracts and supernatants can influence the zeta potential of nanomaterials [2]. This behavior may be associated with the adsorption of biomolecules derived from metabolites of *L. rhamnosus* ATCC 9595 and *L. paracasei* UFTM 2.9, which can partially screen surface charges and modify the electrical environment at the nanoparticle interface. In addition, organic and polymeric species are known to affect the distribution of ions near the slipping plane, influencing the measured zeta potential [42]. In this context, the results reflect a moderately charged surface and provide information on the dispersion behavior of the nanostructures under the experimental conditions employed in this study. These characteristics suggest that electrostatic interactions alone are not sufficient to ensure strong long-term colloidal stability, and that additional stabilization mechanisms may also contribute to particle dispersion behavior.

**Figure 4:**
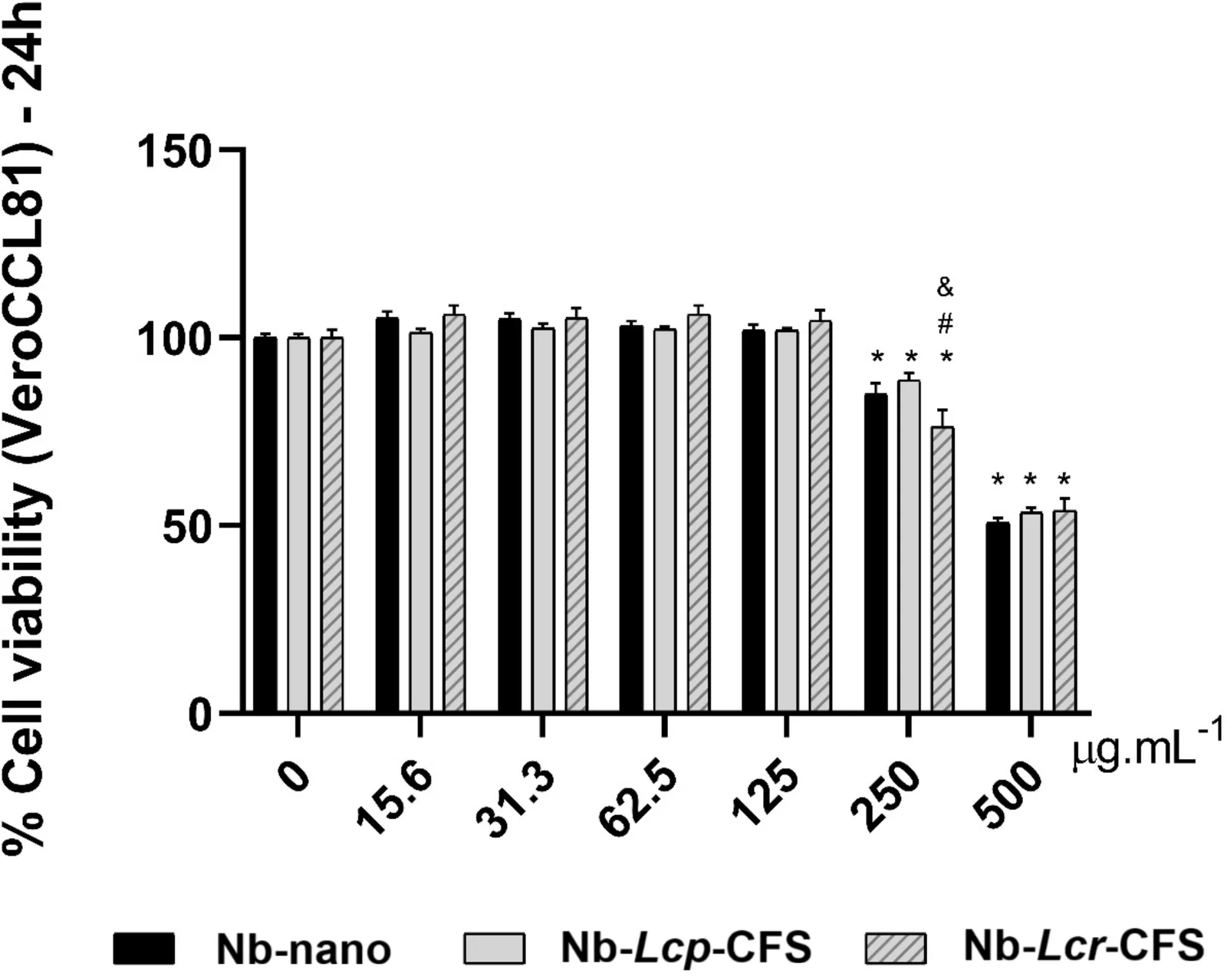
Effects of niobium-based nanostructures on the cell viability. Cell viability (%) of Vero-CCL81 treated with different concentrations of Nb-nano, Nb-*Lcr*-CFS and Nb-*Lcp*-CFS. “*” indicates significant differences compared to non-treated cells, “#” indicates significant differences compared to the Nb-nano at the same concentration and “&” indicates significant differences compared to the Nb-*Lcp*-CFS at the same concentration, according to Tukey’s multiple comparisons test (*p* < 0.05). Triplicates of each of the concentrations from three different assays were performed.

The analysis of in vitro cytotoxic effects is the starting point for evaluating the potential application of newly synthesized nanomaterials for biomedical applications. In this study, Vero-CCL81 cells, one of the most widely used cell lines for toxicological screening, were exposed for 24 h to the biosynthesized niobium-based nanostructures, and cell viability was quantified using the neutral red uptake assay. Cells treated with 500 µg·mL^−1^ of Nb-*Lcr*-CFS and Nb-*Lcp*-CFS exhibited viability values of 53.9 ± 3.3% and 53.6 ± 1.1%, respectively. At 250 µg·mL^−1^, viability increased to 76.3 ± 4.5% for Nb-*Lcr*-CFS and 88.7 ± 1.9% for Nb-*Lcp*-CFS. These data indicate that, at these concentrations, 24-hour treatment significantly reduced cell viability compared with the untreated control (100%). A similar trend was observed for niobium nanostructures synthesized in the absence of LAB-CFS (Nb-nano), with viability values of 50.7 ± 1.4% and 79.2 ± 4.7% following treatment with 500 and 250 µg·mL^−1^, respectively. In addition, Nb-*Lcp*-CFS displayed lower cytotoxicity compared with Nb-*Lcr*-CFS at 250 µg·mL^−1^ (Figure 4), suggesting that the specific metabolites present in the *Lc. paracasei* supernatant may confer a more biocompatible surface profile to the resulting nanostructures.

The 50% cytotoxic concentration (CC_50_), defined as the concentration required to reduce cell viability by 50%, was determined using the complete dose-response dataset obtained across the tested concentration range (0–500 µg·mL^−1^) and estimated through linear regression analysis from cytotoxicity assay data. Vero-CCL81 cells exposed to Nb-nano, for 24 h, exhibited a CC_50_ of 528.0 ± 34.07 µg·mL^−1^, whereas cells treated with Nb-*Lcr*-CFS and Nb-*Lcp*-CFS showed CC_50_ values of 526.7 ± 78.06 and 577.3 ± 59.49 µg·mL^−1^, respectively. Despite the slight difference, these results suggest that Nb-*Lcp*-CFS is slightly less cytotoxic than both Nb-nano and Nb-*Lcr*-CFS (Table 3).

**Table 3:** Mean cytotoxic concentration (CC_50_) of niobium-based nanostructures.

Compounds	CC_50_ (µg·mL^−1^)	*R* ^2^

Nb-nano	528.0 ± 34.1	0.96
Nb-*Lcp*-CFS	526.7 ± 78.1	0.96
Nb-*Lcr*-CFS	577.3 ± 59.5	0.93

The assessment of the toxic effects of newly developed nanomaterials is a fundamental practice to ensure their safe application and to prevent undesirable impacts on biological impacts on biological systems. Maheswaran et al., investigated metal nanoparticles functionalized with medicinal extracts via green synthesis and reported a reduction of cell viability of more than 30% after 72 h of exposure at concentrations above 6.25 µg·mL^−1^ [43]. According to ISO 10993-5 standards [44], a compound is considered cytotoxic when it induces a viability reduction greater than 30%. Our data demonstrated that niobium-based nanostructures synthesized with LAB supernatants induced a viability reduction exceeding this threshold only at the highest concentration tested (500 µg·mL^−1^), indicating comparatively lower cytotoxicity within the evaluated concentration range.

Biocompatibility can also be established through the mean cytotoxic concentration (CC_50_), where higher values indicate lower toxicity of the tested material. However, there is no universal consensus regarding the ideal CC_50_ range that ensures the non-toxicity of a compound as this parameter depends on several variables, including cell types used in the assays. Ferreira et al. considered that, for Vero cells, a CC_50_ value above 100 µg·mL^−1^ characterizes a non-toxic substance [45], whereas Osorio et al. classified compounds within the range of 100–1000 µg·mL^−1^ as moderately cytotoxic [46]. Studies involving silver-doped nanoparticles have reported CC_50_ values around 100 µg·mL^−1^ [47]. Considering these references, the niobium-based nanocomposites investigated in this study, particularly Nb-*Lcp*-CFS, which exhibited the highest CC_50_, demonstrate favorable biocompatibility within the tested biological system.

Several mechanisms may influence cell viability, however, metallic nanoparticles are known to generate reactive oxygen species (ROS) and increase pro-inflammatory cytokine levels, indicating oxidative stress as a potential mechanism of cell death [48]. As demonstrated by Maheswaran et al., ROS generation can be both time- and dose-dependent [43]. Owing to their broad range of applications, niobium-based nanostructures combined with antioxidants have shown good biocompatibility and low cytotoxicity [6]. Niobium pentoxide powder exhibited moderate anti-leishmanial activity and antitumor effects against breast cancer cells [49]. Furthermore, niobium-coated surfaces for biomedical implants did not induce toxic responses related to cell adhesion, proliferation, or viability, indicating that Nb coatings are biocompatible [50]. Overall, these results suggest that niobium-based nanostructures represent a promising and biocompatible platform to be explored.

Regarding the antibacterial potential, the results revealed that the biosynthesized nanostructures obtained using *Lcr*-CFS and *Lcp*-CFS exhibited a MIC value of 1,250 µg·mL^−1^ and a MBC of 2,500 µg·mL^−1^ against *E. coli* (Table 4), indicating that both materials inhibited bacterial growth at relatively high concentrations and exhibited bactericidal activity under the tested conditions. Importantly, at the concentration used during the green synthesis process (10% v/v), neither of the two cell-free supernatants exhibited inhibitory activity against *E. coli*, indicating that the antimicrobial effect arises from the synthesized nanostructures rather than from residual metabolites in the CFS. For comparison, Nb-nano exhibited a MIC of 625 µg·mL^−1^ and an MBC of 2,500 µg·mL^−1^, suggesting that the presence of LAB-derived metabolites may influence the antimicrobial potency of the resulting nanomaterials.

**Table 4:** Minimum inhibitory concentration (MIC) and minimum bactericidal concentration (MBC) of niobium-based nanostructures against *Escherichia coli* ATCC 25922.

Compounds	MIC (µg·mL^−1^)	MBC (µg·mL^−1^)

Nb-nano	625	2,500
Nb-*Lcr*-CFS	1,250	2,500
Nb-*Lcp*-CFS	1,250	2,500

**Figure 5:**
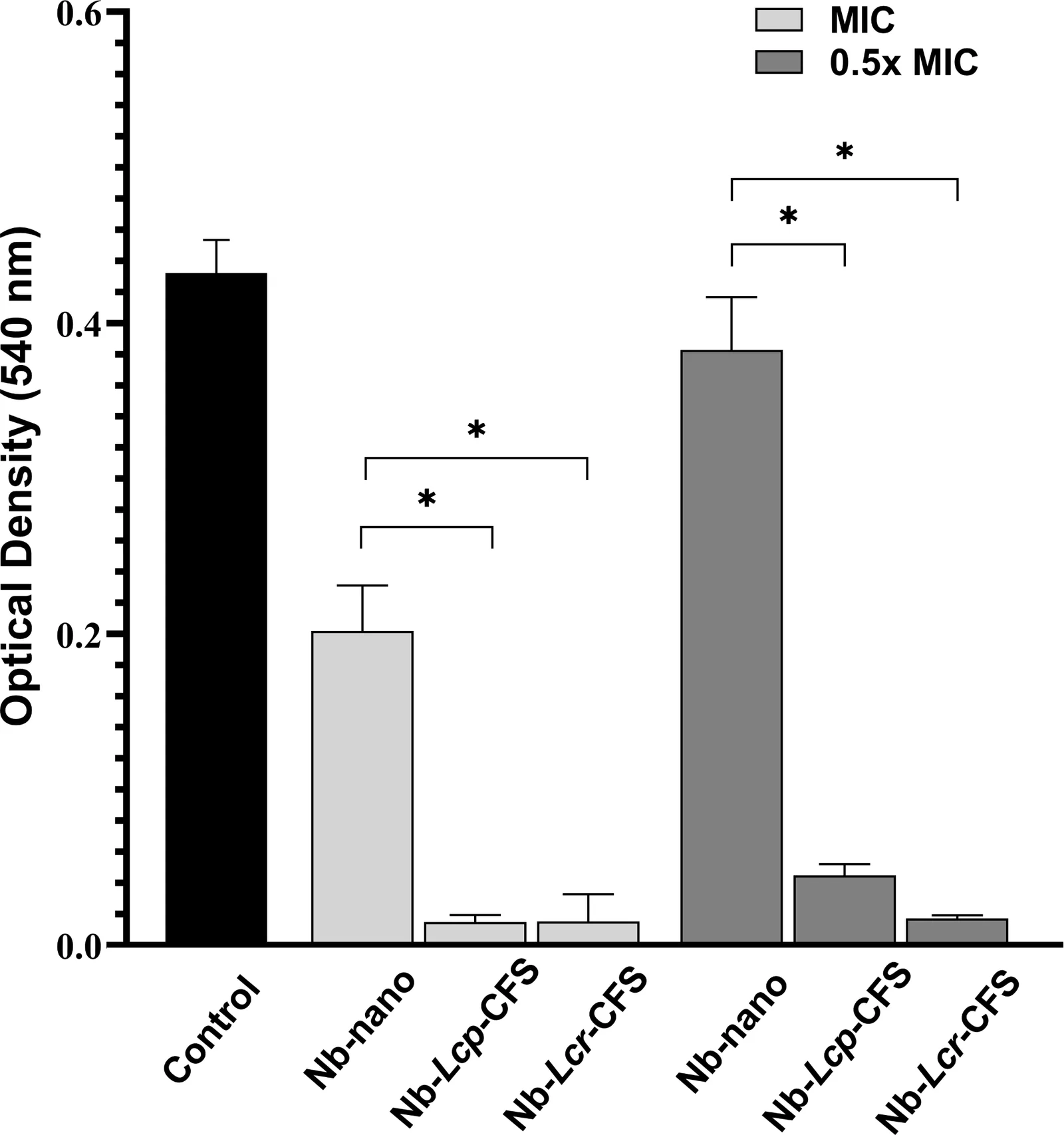
Effect of niobium-based nanostructures (MIC and 0.5 × MIC) on the reduction of *Escherichia coli* ATCC 25922 biofilm formation. “*” indicates significant differences, compared to the Nb-nano at the same concentration, according to Tukey's multiple comparisons test (*p* < 0.05).

When evaluated against *E. coli* biofilm, nanostructures biosynthesized by *Lcr*-CFS and *Lcp*-CFS significantly reduced biofilm formation compared to untreated control and Nb-nano (*p* < 0.05) (Figure 5). At their respective MIC values, Nb-*Lcr*-CFS and Nb-*Lcp*-CFS inhibited biofilm formation by more than 95% (96.3% and 96.4%, respectively). Even at 0.5 × MIC, inhibition remained high, reaching 95.8% for Nb-*Lcr*-CFS and 89.2% for Nb-*Lcp*-CFS. In contrast, Nb-nano reduced *E. coli* biofilm formation by only 51.6% at MIC. The findings demonstrate that the niobium nanostructures biosynthesized in the presence of cell-free supernatants from *Lc. rhamnosus* ATCC9595 and *Lc. paracasei* UFTM 2.9 enhanced antibiofilm activity relative to nanostructures produced without bacterial metabolites. Importantly, at the concentration used during green synthesis (10% v/v), neither of the two cell-free supernatants alone exhibited antibiofilm activity against *E. coli*.

Hamed et al., using supernatants from two actinomycete strains for the green biosynthesis of silver nanoparticles, reported significant antimicrobial activity toward pathogenic microbes, especially *Pseudomonas aeruginosa* and *Enterobacter cloacae*, besides high biofilm inhibition activity against *P. aeruginosa*, *Bacillus subtilis*, and *Staphylococcus aureus*. However, those nanoparticles displayed moderate biofilm inhibition activity against *E. coli*, with a biofilm inhibitory ratio of 67% [51]. Selem et al. synthesized silver nanoparticles using *Aloe vera* leaf extract and reported significant antibacterial (MIC of 85 µg·mL^−1^ and MBC of 127.5 µg·mL^−1^) and antibiofilm activities against *E. coli* uropathogenic (reduction of 55% of biofilm formation at MIC value) [52]. Elabbasy and colleagues reported the antibacterial and antibiofilm activity of green-synthesized zinc oxide nanoparticles using *Stevia rebaudiana* against *E. coli* [53]. According to the authors, the effects were strain- and dose-dependent, and specifically for biofilm, the highest inhibition rates (approximately 80–85%) were observed at concentrations equivalent to 10 × MIC.

In our study, niobium-based nanostructures synthesized using *Lcr*- and *Lcp*-CFS exhibited a pronounced antibiofilm activity against *E. coli*. These findings suggest that these nanostructures may interfere with biofilm development through mechanisms distinct from those responsible for planktonic cell inhibition. Biofilm formation is a complex process involving initial adhesion, cell aggregation, extracellular polymeric substance (EPS) production, and biofilm maturation. Nanostructures may impair these processes by interacting with bacterial cell surfaces, altering physicochemical properties involved in adhesion, and disrupting cell–cell communication required for biofilm organization. In addition, the presence of LAB-derived metabolites during the biosynthesis process may contribute to the enhanced antibiofilm performance by modifying the nanostructure surface or providing additional bioactive components capable of interfering with EPS production, cell–cell communication, and biofilm stability. The marked inhibition observed even at sub-MIC concentrations indicates that the antibiofilm effect is not solely dependent on bacterial growth inhibition, but rather involves interference with biofilm-associated processes. Therefore, Nb-*Lcr*-CFS and Nb-*Lcp*-CFS represent promising materials for controlling *E. coli* biofilms.

## Conclusion

To the best of our knowledge, this study is the first to demonstrate the ability of cell-free supernatants from *Lc. rhamnosus* ATCC 9595 and *Lc. paracasei* UFTM 2.9 to simultaneously act as reducing, capping, and stabilizing agents for the green synthesis of bioactive niobium oxide nanostructures. Structural characterization confirmed the successful formation of niobium oxide nanostructures incorporating CFS-derived metabolites, resulting in materials with characteristics consistent with hydrated niobium pentoxide and predominantly amorphous Nb_2_O_5_ structures with aggregated and irregular morphologies. The incorporation of LAB-derived metabolites during synthesis represents a key feature of this approach as these biomolecules may contribute to the surface functionalization and enhanced biological performance of the resulting nanostructures. Biological evaluation demonstrated that the biosynthesized nanostructures exhibited moderate antibacterial activity against planktonic *E. coli* cells and pronounced antibiofilm efficacy, with biofilm inhibition exceeding 95% under specific conditions. This pronounced antibiofilm effect, particularly observed at subinhibitory concentrations, suggests that the nanostructures interfere with biofilm-associated processes beyond simple bacterial growth inhibition. In addition, the low cytotoxicity observed toward mammalian cells supports their potential as safer bioactive materials. Collectively, these findings highlight the role of LAB-derived metabolites as biological mediators for the development of niobium-based nanostructures with enhanced antibiofilm properties and demonstrate the potential of this sustainable synthesis strategy for generating innovative materials for bacterial biofilm control.

## Experimental

### Microorganisms, growth conditions, and cell-free supernatant obtention

*Lacticaseibacillus rhamnosus* ATCC 9595 was kindly provided by Fiocruz (Fundação Oswaldo Cruz, Brazil), and *Lacticaseibacillus paracasei* UFTM 2.9 was previously isolated from unpasteurized milk by our research group. Bacteria were kept preserved at −20 °C in 20% glycerol-containing brain heart infusion (BHI) broth. Both strains show the ability to survive in the presence of bile salts and acidic pH and exhibit antagonistic activity against spoilage and pathogenic bacteria [19]. Analysis of the complete genome of these two bacterial strains revealed genes related to probiotic properties and the absence of virulence genes or antimicrobial resistance determinants, revealing a promising safety profile [54].

*Lc. rhamnosus* ATCC 9595 and *Lc. paracasei* UFTM 2.9 were grown in Man–Rogosa–Sharpe (MRS) broth (Kasvi, Spain), at 37 °C for 24 h, under microaerophilic conditions. A stationary-phase culture of the bacterial strains was centrifuged (12,500 rpm, 15 min), and the supernatant was filtered through a 0.22 μm cellulose nitrate membrane filter (Kasvi) to obtain the cell-free supernatants of *Lc. rhamnosus* ATCC 9595 (*Lcr*-CFS) and *Lc. paracasei* UFTM 2.9 (*Lcp*-CFS). The cell-free supernatants (CFS) were stored at −20 °C until use.

### Synthesis of nanostructured niobium-based oxides

Niobium pentachloride (NbCl_5_) was used as a precursor for the synthesis of niobium-based nanostructures. Initially, the precursor at a concentration of 25 mg·mL^−1^ was rapidly added to deionized water containing 10% (v/v) of *Lcr*-CFS or *Lcp*-CFS, promoting its hydrolysis in the reaction medium. Subsequently, the reaction mixture was magnetically stirred for 24 h at 30 °C, protected from light and without additional pH control. Subsequently, the mixture was centrifuged and washed with deionized water at 4,000 rpm for three cycles of 15 min each. Finally, the resulting pellet was dried in an oven at 50 °C overnight and then macerated until it formed a fine powder [6,55]. As a final result of the process, niobium-based nanostructures were synthesized: Nb-*Lcr*-CFS and Nb-*Lcp-*CFS. The control material (Nb-nano) was synthesized following the same procedure described above for CFS-mediated systems, but in the absence of CFS. All other conditions (precursor concentration, stirring time, temperature, washing, and drying) were maintained [6].

### Optical and structural characterization of the nanostructures

UV–vis spectra were acquired using a Shimadzu UV-1800 spectrophotometer, operating within the wavelength range of 190 to 500 nm. For this purpose, the samples were prepared as a stock solution at a concentration of 2 mg·mL^−1^ in deionized water and exposed to an ultrasonic bath for 1 h. As a dispersion medium, a 1% solution of polysorbate 80 (Tween 80) in deionized water was used [6]. FTIR analyses were performed using a Bruker ALPHA II spectrometer (Massachusetts, USA). Measurements were made in the spectral range of 4,000 to 400 cm^−1^, with a resolution of 4 cm^−1^. X-ray diffraction (XRD) patterns were obtained using a Shimadzu XRD-6100 diffractometer (Kyoto, Japan), equipped with Cu Kα radiation, operating at 40 kV and 30 mA. Data were collected over a 2θ range of 5° to 80°.

### Morphological characterization of the nanostructures

Scanning-transmission electron microscopy (STEM) analysis was performed using an FEI Magellan 400 L electron microscope equipped with a field-emission gun and a STEM detector operating at an accelerating voltage of 5 kV. Samples were prepared by depositing a drop of the niobium-based nanostructured suspension, previously dispersed in isopropyl alcohol, onto an aluminum grid. Prior to deposition, the suspensions were exposed to an ultrasonic bath for 10 min. Subsequently, the samples were dried in an oven at 60 °C. Additionally, a detector in the lens was used to collect secondary electrons to improve image resolution and contrast. Scanning electron microscopy (SEM) analyses were performed using a TESCAN VEGA 3 LMU microscope operating at 20 kV, coupled with an energy-dispersive X-ray (EDX) spectrometer. The samples were coated with a thin layer of gold via sputtering to enhance their electrical conductivity prior to analysis. Micrographs were acquired in both secondary electron and backscattered electron modes [6,56].

### Hydrodynamic diameter and charge properties of nanostructures

Hydrodynamic diameter, polydispersity index (PDI), and zeta potential of the nanostructures were determined using a ZetaSizer Nano ZS90 instrument (Malvern Instruments Ltd., UK). Dynamic light scattering (DLS) measurements were performed to evaluate the hydrodynamic size and PDI, while zeta potential analysis was conducted to assess the surface charge and colloidal stability of the synthesized systems. For analysis, samples were diluted in deionized water containing 1% Tween 80 and subjected to ultrasonic treatment. The measurements were performed in a 1% (w/v) Tween 80 aqueous solution (pH 5.5) to improve the dispersion of the intrinsically hydrophobic particles and minimize aggregation. As a non-ionic surfactant, Tween 80 provides steric stabilization, thereby improving the colloidal system and enabling reliable electrophoretic mobility measurements. Measurements were conducted in DTS 1060 cuvettes (Malvern) equipped with gold electrodes, employing Doppler velocimetry to determine the zeta potential [6,57–58].

### Evaluation of the toxicity of the niobium-base nanostructures

The cytotoxic and biocompatibility evaluation of niobium-based nanostructures was performed in accordance with ISO 10993-5:2009, which establishes procedures for testing materials intended for potential biomedical applications [44]. Vero CCL-81 cells, listed in ISO among the cell lines suitable for cytotoxicity testing, were employed as an appropriate model for the initial screening of cytotoxicity and biocompatibility due to their robust growth characteristics, reproducibility, and sensitivity to cytotoxic agents, making them a widely accepted model for preliminary safety assessment of novel biomaterials. Initially, the niobium-based nanomaterials were sterilized by exposure to UV irradiation (254 nm) for 30 min, followed by immersion in a water bath at 63 °C for 60 min. Subsequently, samples were resuspended in DMSO at a final concentration of 10 mg·mL^−1^. Vero-CCL81 cells were seeded in 96-well plates at a density of 10^4^ cells per well and incubated for 24 h under controlled conditions (37 °C, 5% CO_2_). The cells were then exposed for 24 h to various concentrations of the nanoformulations (0–500 µg·mL^−1^), while isolated LAB supernatants were tested at concentrations corresponding to 10% of the nanostructure concentration. Cell viability was assessed using the neutral red uptake assay, which is based on the incorporation of the vital dye into lysosomes of viable cells through hydrophobic and electrostatic interactions. Structural damage to the plasma membrane proportionally reduces dye uptake, enabling differentiation between viable, damaged, and dead cells [59]. After exposure, the dye retained in cells was quantified by measuring absorbance at 540 nm using a microplate reader (ThermoPlate TpReader, USA) [60]. Results were expressed as the percentage of viable cells relative to untreated controls. Cytotoxicity curves were generated by plotting cell viability against sample concentration, and the 50% cytotoxic concentration (CC_50_), defined as the concentration that causes a 50% reduction in cell viability, was determined by linear regression using GraphPad Prism 8.0 and Excel softwares. All experiments were performed in triplicate and independently repeated at three times to ensure reproducibility.

### Interference of nanostructured systems on bacterial growth and biofilm formation

The antimicrobial potential of the niobium-based nanostructures and isolated CFSs was assessed against *Escherichia coli* ATCC 25922 using the broth microdilution method. Initially, different concentrations of nanostructures (2,500 to 156.25 µg·mL^−1^; 50 μL) or CFSs (10%; 50 μL) were added to 96-well polystyrene microplates containing BHI broth (50 μL). Subsequently, a stationary-phase bacterial suspension standardized to 0.5 McFarland (10 μL; 10^6^ CFU·mL^−1^) was added to each well, and plates were incubated at 37 °C for 24 h under aerobic conditions. Negative controls contained uninoculated BHI broth, while positive controls consisted of medium inoculated with *E. coli* in the absence of tested agents. The minimum inhibitory concentration (MIC) was defined as the lowest concentration of the material that completely inhibited visible bacterial growth [61]. The minimum bactericidal concentration (MBC) was determined by subculturing aliquots (5 μL) from wells corresponding to concentrations equal to or higher than the MIC onto BHI agar plates without the tested compounds. The plates were incubated at 37 °C for 24 h under aerobic conditions. The MBC was defined as the lowest concentration that completely inhibited bacterial growth after subculturing [61].

The effect of the nanostructures and CFSs on *E. coli* biofilm formation was evaluated using the crystal violet assay. Briefly, stationary-phase bacterial suspensions standardized to 0.5 McFarland (10 μL) were inoculated into 96-well microplates containing BHI broth (2×; 50 μL) and exposed to nanostructures (2,500 to 156.25 µg·mL^−1^; 50 μL) or CFSs (10%; 50 μL). Plates were incubated at 37 °C for 24 h under aerobic conditions. After incubation, the wells were rinsed with distilled water (three times) and stained with crystal violet solution (2%; 150 μL) for 15 min at room temperature. Excess stain was removed, and the bound dye was solubilized with ethanol (95%; 150 μL). The absorbance was measured at 540 nm using a microplate reader to quantify biofilm biomass [62].

### Statistical analysis

Statistical analyses were performed using Excel for Windows (Microsoft, USA) and GraphPad Prism^®^ software version 8.0 (GraphPad, Inc., La Jolla, CA, USA). The normality of data distribution was verified and confirmed to follow a Gaussian pattern. Based on this, two-way ANOVA was applied for group comparisons, followed by Tukey’s multiple comparison test for cell viability analysis and to assess the effect of niobium-based nanostructures on biofilm formation. All results were expressed as mean ± standard deviation, and statistical significance was considered at *p* < 0.05.

## Supporting Information

File 1 Additional figure.

## Data Availability

All data that supports the findings of this study is available in the published article and/or the supporting information of this article.
