# Supplementary material for: Biosynthesis, characterization, and biofunctional assessment of niobium oxide nanostructures obtained using lactic acid bacteria-derived supernatants
Source: Beilstein J Nanotechnol. 2026 Aug 17;17:1128–41. doi: 10.3762/bjnano.17.77 (PMC13501231; doi:10.3762/bjnano.17.77)
Supplement: Supplementary file 1 [file Beilstein_J_Nanotechnol-17-1128-s001.pdf]

BEILSTEIN JOURNAL OF NANOTECHNOLOGY

Supporting Information

for

Biosynthesis, characterization, and biofunctional assessment of niobium oxide nanostructures obtained using lactic acid bacteria-derived supernatants

Pablo A. Oliveira, Gabriella T. L. Teixeira, Pollyane S. Oliveira, Thaís S. Farnesi-de-Assunção, Isabela S. Rotta, Karina F. D. Vicentine, Dayane S. Alvares, Jéferson A. Moreto, Aline D. Paiva and Natália B. L. Slade

Beilstein J. Nanotechnol. **2026,** *17,* 1128–1141. doi:10.3762/bjnano.17.77

Additional figure

License and Terms: This is a supporting information file under the terms of the Creative Commons Attribution License (https://creativecommons.org/licenses/by/4.0). Please note that the reuse, redistribution and reproduction in particular requires that the author(s) and source are credited and that individual graphics may be subject to special legal provisions.
The license is subject to the *Beilstein Journal of Nanotechnology* terms and conditions: (https://www.beilstein-journals.org/bjnano/terms)

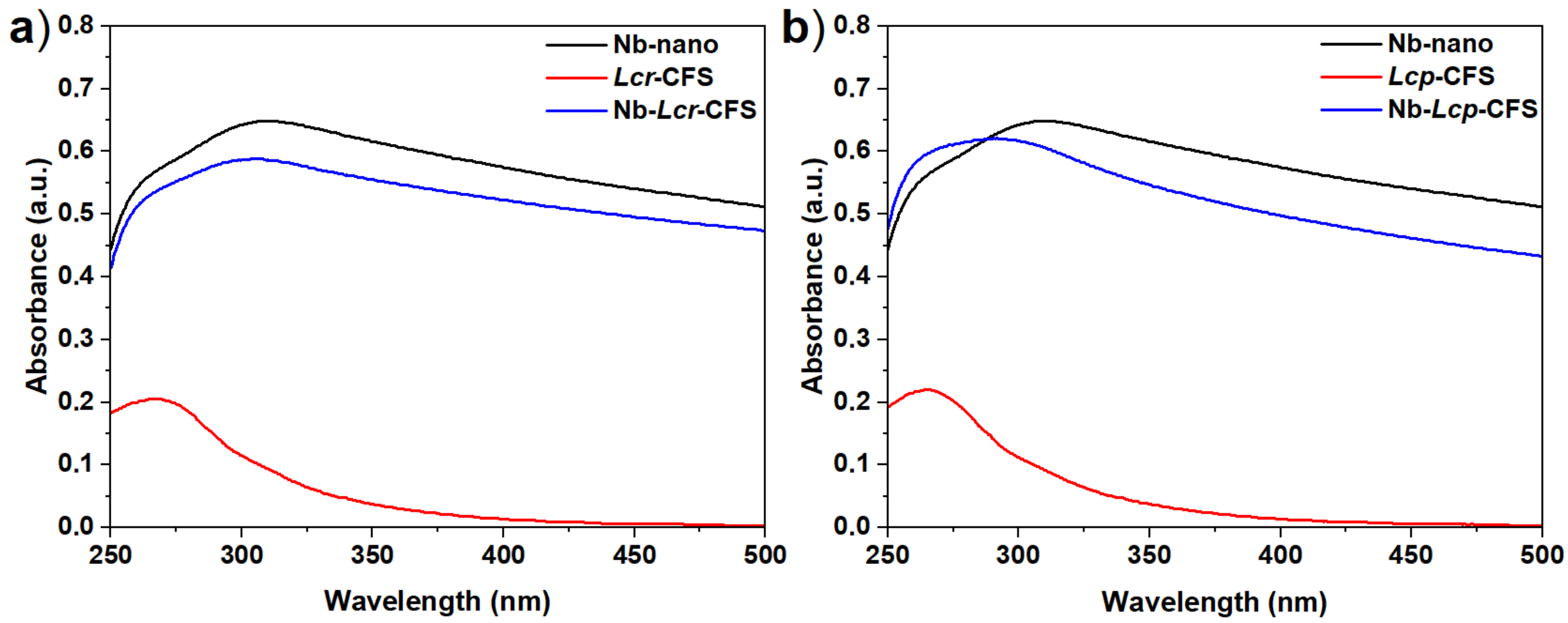

Figure S1: UV–Vis spectra of (A) Nb-*Lcr*-CFS and 10% (v/v) *Lcr*-CFS, and (B) Nb-*Lcp*-CFS and 10% (v/v) *Lcp*-CFS. The spectrum of the control niobium-based nanostructures synthesized in the absence of LAB-CFS (Nb-nano) is included for comparison. The Nb-nano, Nb-*Lcr*-CFS, and Nb-*Lcp*-CFS samples are presented at a concentration of 0.37 mg·mL^{-1}.
